# Coronary crossing

**DOI:** 10.1007/s12471-020-01524-9

**Published:** 2020-11-30

**Authors:** J. J. D. de Jong, H. Tent

**Affiliations:** 1 Medisch Spectrum Twente, Thorax Center, Enschede, The Netherlands; 2 Martini Hospital, Groningen, The Netherlands

**Fig. 1:**
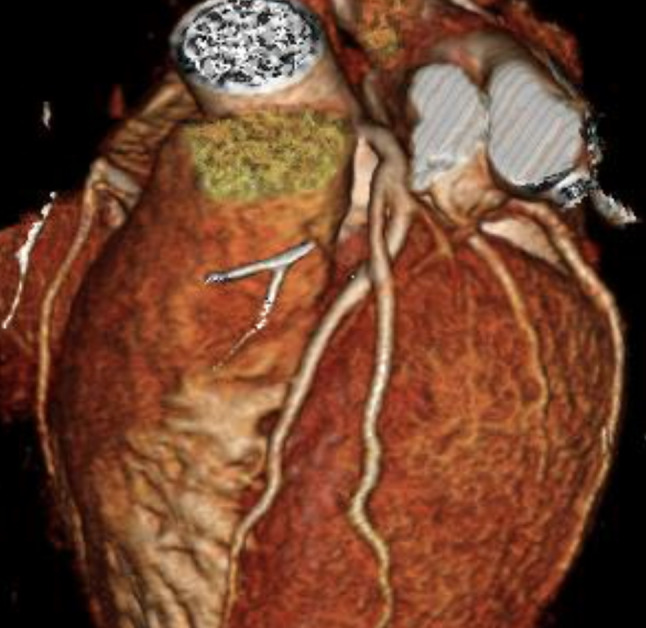
Crossing between a diagonal branch and the left anterior descending artery. The diagonal branch is seen branching off from the left main coronary artery, then crossing over the left anterior descending artery to follow its usual course

A 46-year-old male was sent to our outpatient clinic because of an abnormal exercise stress test, which was performed for sports screening. He was asymptomatic. Coronary computed tomography angiography showed no atherosclerotic coronary artery disease. However, a crossing was seen of a diagonal branch with the left anterior descending artery (Fig. 1). There was no compression of either vessel. Therefore, the crossing was considered to be benign and the exercise test to be false-positive.

The epicardial crossing of coronary arteries has been published in only a dozen of cases worldwide and is still considered to be impossible by many. Whether coronary crossing is a rarity or it is often overlooked, is debatable. Back in 1985, Muyldermans et al. reported a case series from their own catheterisation laboratory [1]. The crossing of coronary arteries was considered pathological in none of the published cases, which may also explain the low incidence of reporting.
